# Surface Reconstruction of Silicone-Based Amphiphilic Polymers for Mitigating Marine Biofouling

**DOI:** 10.3390/polym16111570

**Published:** 2024-06-01

**Authors:** Chuanying Wei, Yan Zhang, Zhen Tang, Changan Zhang, Jianhua Wu, Bo Wu

**Affiliations:** Xiamen Key Laboratory of Marine Corrosion and Intelligent Protection Materials, School of Marine Engineering, JiMei University, Xiamen 361021, China; 202114824002@jmu.edu.cn (C.W.); m17859720856@163.com (Y.Z.); 18363870097@163.com (Z.T.); 202212855046@jmu.edu.cn (C.Z.)

**Keywords:** silicone-based, amphiphilic, surface reconstruction, hydration layer, antifouling

## Abstract

Poly(dimethylsiloxane) (PDMS) coatings are considered to be environmentally friendly antifouling coatings. However, the presence of hydrophobic surfaces can enhance the adhesion rate of proteins, bacteria and microalgae, posing a challenge for biofouling removal. In this study, hydrophilic polymer chains were synthesised from methyl methacrylate (MMA), Poly(ethylene glycol) methyl ether methacrylate (PEG-MA) and 3-(trimethoxysilyl) propyl methacrylate (TPMA). The crosslinking reaction between TPMA and PDMS results in the formation of a silicone-based amphiphilic co-network with surface reconstruction properties. The hydrophilic and hydrophobic domains are covalently bonded by condensation reactions, while the hydrophilic polymers migrate under water to induce surface reconstruction and form hydrogen bonds with water molecules to form a dense hydrated layer. This design effectively mitigates the adhesion of proteins, bacteria, algae and other marine organisms to the coating. The antifouling performance of the coatings was evaluated by assessing their adhesion rates to proteins (*BSA-FITC*), bacteria (*B. subtilis* and *P. ruthenica*) and algae (*P. tricornutum*). The results show that the amphiphilic co-network coating (e.g., P-AM-15) exhibits excellent antifouling properties against protein, bacterial and microalgal fouling. Furthermore, an overall assessment of its antifouling performance and stability was conducted in the East China Sea from 16 May to 12 September 2023, which showed that this silicon-based amphiphilic co-network coating remained intact with almost no marine organisms adhering to it. This study provides a novel approach for the development of high-performance silicone-based antifouling coatings.

## 1. Introduction

Marine biofouling refers to the undesirable accumulation of microorganisms, algae and macrofouling organisms (e.g., barnacles, mussels, etc.) that attach to marine structures [1,2,3]. This process results in increased hull resistance to navigation, increased maintenance costs, increased fuel consumption and greenhouse gas emissions, and potential species invasions with consequent impacts on local ecosystems [4]. Antifouling coatings are recognised as one of the most cost-effective strategies to combat marine fouling [5,6], with fouling release coatings (FRCs) being non-toxic, environmentally friendly coatings that use hydrodynamics to prevent macrofouling organisms from adhering to their surfaces [7,8,9,10].

Poly(dimethylsiloxane) (PDMS) is a highly effective FRC due to its low surface energy and modulus of elasticity [8,11], which facilitates the efficient removal of macrofouling organisms such as barnacles by seawater washing. However, the effectiveness of PDMS-based coatings in preventing fouling organism settlement is limited by non-specific binding effects. In particular, PDMS exhibits inadequate resistance to the accumulation and growth of marine slime layers composed of bacteria, algae and the secretion of extracellular polymers [12,13]. Researchers have found that the formation of a hydrated layer by hydrophilic polymers (e.g., PEG-based coatings) effectively prevented fouling organisms from settling in the early stages [14]. However, once attached to the surface, the release of fouling organisms became difficult due to the properties of the coating [15]. Therefore, a more promising antifouling strategy is to create amphiphilic polymers by physically mixing or chemically crosslinking hydrophilic and hydrophobic polymers [16,17].

The amphiphilic modification of PDMS is the most commonly used strategy to improve its antifouling ability [18]. However, due to the thermodynamic incompatibility between hydrophobic and hydrophilic regions, this can lead to the macroscopic phase separation of PDMS from the hydrophilic polymer, compromising material stability [19]. Therefore, the formation of silicon-based amphiphilic co-network via a forced chemical crosslinking approach is an effective solution to address this issue. Zhao et al. [20] prepared a PVP-based hydrophilic polymer in which an antifouling silicone-based amphiphilic polymer for marine applications with excellent mud resistance was obtained by crosslinking between the reactive hydroxyl functional group of 2-hydroxyethyl methacrylate (HEMA) and PDMS. The use of silane coupling agents containing both siloxane groups and reactive functional groups as crosslinking monomers is an innovative strategy. Guo et al. [21] and Zeng et al. [22] successfully synthesised two novel silicone-based amphiphilic polymers using 3-isocyanatopropyltriethoxysilane and 3-mercaptopropyltrimethoxysilane, respectively, which exhibited exceptional antifouling properties. HEMA forms hydrophilic chains with antifouling active monomers through radical polymerisation, which can effectively increase the crosslink density of the crosslinked network. The hydrophilic polymers are supplemented with silane coupling agents as capping agents to facilitate crosslinking with PDMS, and dehydration condensation between silanols promotes the crosslinking reaction. The incorporation of crosslinking monomers is therefore essential for the preparation of silicone-based amphiphilic co-network.

**Scheme 1:**
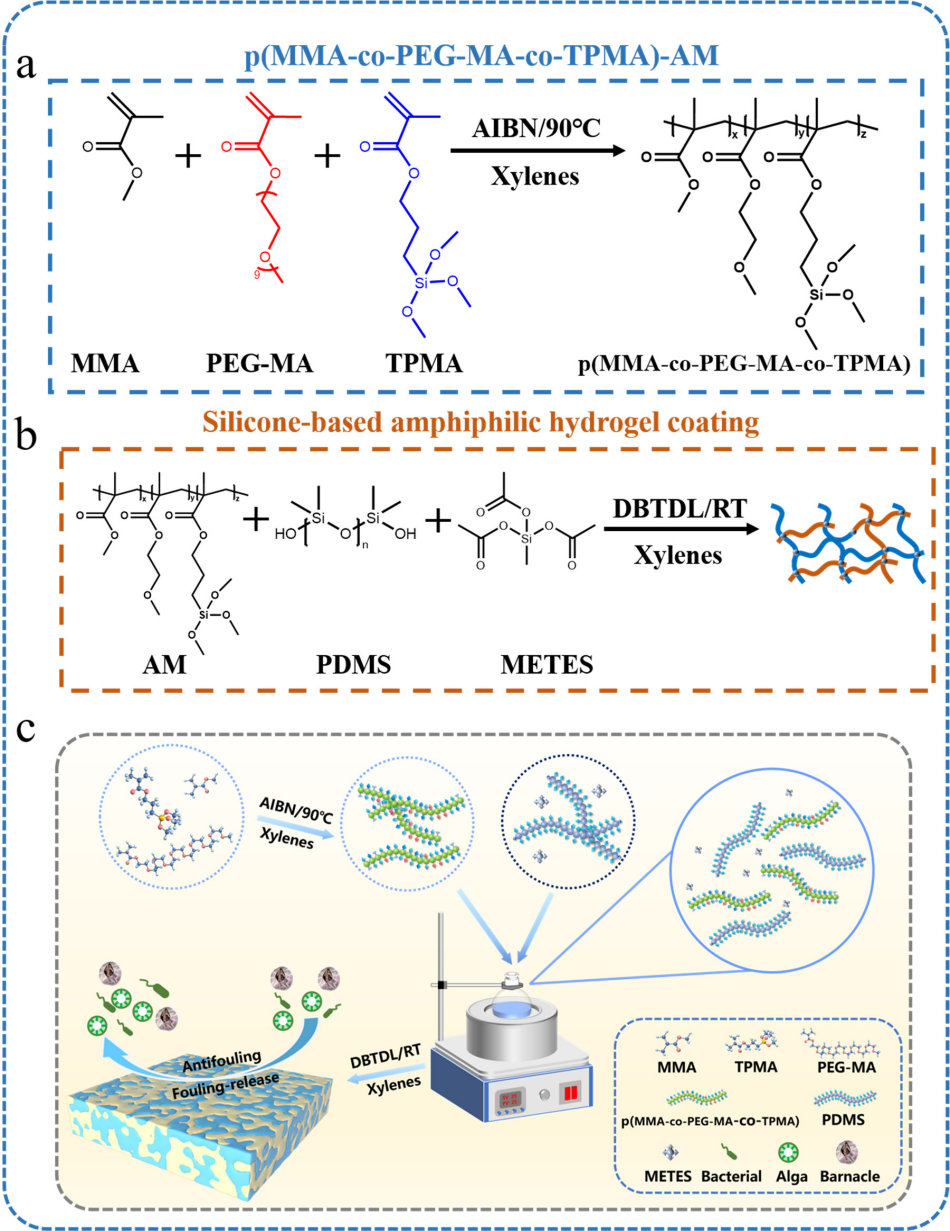
(**a**) Synthesis of poly(MMA-co-PEG-MA-co-TPMA). (**b**) The synthesis route of the silicone-based amphiphilic co-networks coating. (**c**) Synthetic scheme.

In order to develop amphiphilic co-network polymers with anti-adhesive properties, we selected Poly(ethylene glycol) methyl ether methacrylate (PEG-MA) as a hydrophilic monomer due to its low interfacial energy (water-PEG, <5 mN/m) [14,23], which allows for the formation of a dense hydration layer with water molecules and imparts excellent anti-adhesive properties to the coatings. In addition, hydroxyl-terminated PDMS (HO-PDMS-OH) was used as the hydrophobic monomer, while 3-(trimethoxysilyl) propyl methacrylate was used as the crosslinker [24]. 3-(trimethoxysilyl) propyl methacrylate has an ethylene linkage and reacts with methacrylate monomer to form polymer chains. Compared with KH590 or other silane coupling agents, it not only forms a crosslinked network by dehydration condensation with PDMS-OH, but also improves the crosslink density and stability of amphiphilic polymers. In this study, we successfully synthesised a silicone-based amphiphilic gel marine antifouling coating incorporating polyethylene glycol (Scheme 1). Firstly, p(MMA-PEG-MA-TPMA) was synthesised using methyl methacrylate (MMA), polyethylene glycol monomethyl ether methacrylate (PEG-MA) and 3-(trimethoxysilyl)propyl methacrylate (TPMA) as reaction monomers, denoted as AM. Subsequently, at ambient temperature, TPMA acts as a crosslinking agent to facilitate the crosslinking of AM, PDMS and methyltriethoxysilane to form a silicone-based amphiphilic gel coating. Finally, the antifouling performance was evaluated in laboratory and natural marine environments. The amphiphilic coating exhibited exceptional antifouling performance in both environments, highlighting its potential as an environmentally friendly marine antifouling coating.

## 2. Materials and Methods

### 2.1. Materials

Hydroxyl-terminated PDMS (HO-PDMS-OH, Mw = 28,000 g/mol) was purchased from Wacker (Shanghai, China) Co., Ltd. Methyl methacrylate (MMA), Poly(ethylene glycol) methyl ether methacrylate (PEG-MA, Mw = 475 g/mol), 3-(trimethoxysilyl)propyl methacrylate (TPMA), 2,2′-azoylsobutyronitrile (AIBN), dibutyltin dilaurate (DBTDL) and fluorescent isothiocyanate-labelled bovine serum albumin (FITC-BSA) were all purchased from Aladdin (Shanghai, China) Co., Ltd. Methyltriacetoxysilane (METES), xylene and n-hexane were purchased from Macklin (Shanghai, China) Co., Ltd. The above reagents were used as received without further purification.

### 2.2. Synthesis of Poly(MMA-co-PEG-MA-co-TPMA)

**Figure 1:**
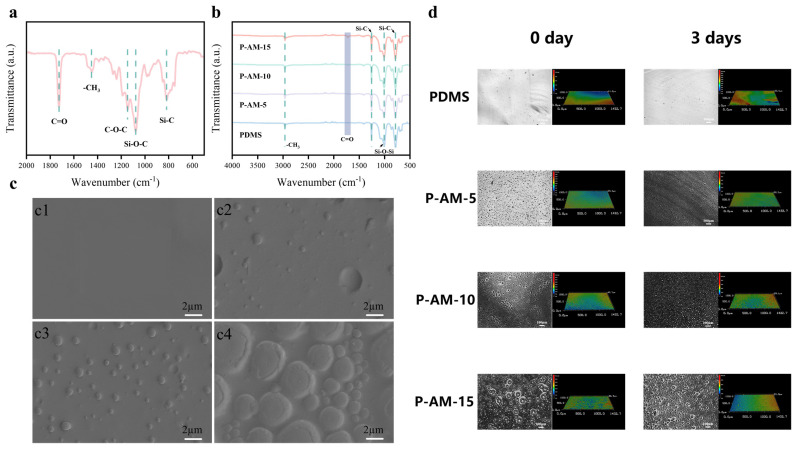
(**a**) FT-IR spectra of the random copolymer poly(MMA-co-PEG-MA-co-TPMA). (**b**) ATR-IR spectra of PDMS and amphiphilic coatings with different additives. (**c**) SEM images of the coating, PDMS (**c1**), P-AM-5 (**c2**), P-AM-10 (**c3**) and P-AM-15 (**c4**). (**d**) The surface morphology images of the coating before and after immersion for 3 days.

As shown in Scheme 1a, non-crosslinked polymer chains were synthesised by radical polymerisation. Briefly, MMA (5 g, 50 mmol), PEG-MA (2.375 g, 5 mmol) and TPMA (7.51 g, 30 mmol) were dissolved in 15 mL of xylenes and degassed with N_2_ for 10 min. The reactants were polymerised at 90 °C for 4 h. The crude product was washed three times with n-hexane to remove residual monomer. It was then subjected to vacuum oven drying at 30 °C overnight and the resulting compound was designated as AM. The results of the analysis using both proton nuclear magnetic resonance (^1^H NMR) spectroscopy and Fourier transform infrared (FT-IR) spectroscopy are shown in Figure S1 and Figure 1a. The molecular mass of the poly (MMA-co-PEG-MA-co-TPMA) was determined by GPC, showing a number average molecular mass (M_n_) of 11,000 g/mol and a weight average molecular mass (M_w_) of 21,000 g/mol, and a polydispersity index (PDI) of 1.81. The AM achieved a yield of 90%.

### 2.3. Coating Preparation

Different amounts of AM (wt%) were mixed with HO-PDMS-OH (5 g) in a mixture of xylenes (10 mL). The mixture was stirred continuously for 30 min at room temperature to form homogeneous solution A. Then, METES (0.5 g) was dispersed in xylene (5 mL) and sonicated for 5 min to form solution B. Subsequently, solution B was gradually added to solution A at room temperature with 30 min of stirring to produce poly(MMA-co-PEGMA-co-TPMA)-l-PDMS, referred to as P-AM coating (Scheme 1b). The P-AM coatings were then prepared as follows. The amphiphilic prepolymer was applied to the epoxy resin/glass sheet, crosslinked and dried for 48 h. P-AM-X coatings were represented by P-AM-5, P-AM-10 and P-AM-15, respectively (the variable “X” represents the mass fraction of AM added). The detailed compositions of these coatings are given in Table S3.

### 2.4. Characterization

^1^H-NMR spectra were obtained using a spectrometer (Bruker Avance NEO 600, Ettlingen, Germany). Polymers were dissolved in deuterated chloroform (CDCl_3_) and chemical shifts were referenced to tetramethylsilane as internal standard and reported in ppm(d). Molecular weight was determined by gel permeation chromatography (Waters GPC 1515, Milford, MA, USA). The reference material used in this study was polystyrene (PS), with tetrahydrofuran (THF) employed as the mobile phase and a flow rate set at 1.0 mL/min. The 3D morphology of the sample surface was analysed using confocal laser scanning microscopy (CLSM), specifically the KEYENCE VK-X250 (KEYENCE, Osaka, Japan). The XYZ fast scanning mode was used to measure the surface morphology of the coating. The microscopic morphology was analysed by SEM analysis (Sigma 500, Zeiss, Germany). Fourier transform infrared spectrometry and attenuated total reflectance infrared spectrometry (FT-IR, ATR-IR, Thermofisher, Waltham, MA, USA) spectra were obtained using a Nicolet iS50 (Thermo, Waltham, MA, USA) with a scan range of 4000–500 cm^−1^.

### 2.5. Contact Angle Measurement

The surface hydrophilicity and hydrophobicity were measured at room temperature by water contact angle measurements *θ_S_* (Theta Flex, Biolin Ltd., Frölunda, Sweden) and the surface energy was determined by calculation based on the contact angles of water and diiodomethane (CH_2_I_2_). Water droplets were measured at 3 µL and diiodomethane at 2 µL. Five different positions were selected for each sample to measure the contact angle and the average value was taken as *θ*. The surface energy was determined using the Owens–Wendt method. The measurements were carried out under standard conditions of room temperature and 50% relative humidity. Two liquids with different surface tensions, water and CH_2_I_2_, were used for the measurements [25], followed by the calculation of the surface energy using the Owens–Wendt method [26] (Equations (1) and (2)) and Young’s Equation (3) [27]:

(1) $$\gamma_{s}=\gamma_{s}^{d}+\gamma_{s}^{p}$$

(2) $$\gamma_{sL}=\gamma_{s}+\gamma_{L}-2{\left(\gamma_{s}^{d}\gamma_{L}^{d}\right)}^{1/2}-{2\left(\gamma_{s}^{p}\gamma_{L}^{p}\right)}^{1/2}$$

(3) $$\left(1+\cos\theta \right)\gamma_{L}={2\left(\gamma_{s}^{d}\gamma_{L}^{d}\right)}^{1/2}+{2\left(\gamma_{s}^{p}\gamma_{L}^{p}\right)}^{1/2}$$

The superscripts *d* and *p* represent the disperse and polar components of the surface free energy, respectively. The solid surface energy is denoted by *γ_S_*, the liquid surface tension by *γ_L_*, *γ_SL_* is the liquid–solid interfacial tension and *θ* is the contact angle at the gas–solid–liquid three-phase interface.

The water dynamic contact angle was measured after the samples were immersed in artificial seawater for 3 days. At room temperature, a drop of water (3 μL) was dropped on the sample and the contact angle was recorded at 0, 1, 3, 5, 7 and 10 min [28]. To reduce the potential error due to evaporation of the water droplet, it is necessary to perform three sets of replicate experiments.

### 2.6. FITC-BSA Adsorption Tests

FITC-BSA solution was prepared using PBS (2 mg/mL). P-AM-x and control PDMS coatings were incubated in the FITC-BSA solution for 2 h in the dark, then thoroughly rinsed with deionised water and observed under an inverted microscope. Protein adsorption on the surface of the coatings was measured using an inverted fluorescence microscope. The fluorescence image is shown in Figure S2.

### 2.7. Bacterial Adhesion Test

*Bacillus subtilis* (*B. subtilis*, Gram-positive) and *Pseudoalteromonas ruthenica* (*P. ruthenica*, Gram-negative), two representative marine bacteria, were selected for bacterial adhesion testing on the coatings. P-AM-x and control PDMS samples were placed in 24-well cell culture plates to which 100 µL of bacterial suspension (10^6^ CFU/mL) was added. After incubation at 37 °C for 48 h, the surface was gently rinsed three times with ASW to remove unattached bacteria. An inverted fluorescence microscope was used to take images of bacterial adhesion on different surfaces under blue light excitation. The fluorescence image is shown in Figure S2.

The surface area covered by *B. subtilis*, *P. ruthenica* and *P. tricornutum* adhering to the PDMS coating was quantified as N_1_. Similarly, the surface area covered by these microorganisms on the P-AM-x coating was measured and recorded as N_2_ [29]. The anti-adhesion rate (R) was calculated using Equation (4):

(4) $$R=\left(1-\frac{N_{2}}{N_{1}}\right)\times 100\%$$

### 2.8. Algal Resistance Tests

*Phaeodactylum tricornutum* (*P. tricornutum*) was selected as the test organism. The samples were first immersed in a marine microalgae culture medium supplemented with 30 mL of algal suspension (5 × 10^6^ cells/mL). The samples were then cultured in a light–dark cycle at 20 °C for 7 days. Finally, the surface was washed with sterile ASW to remove unattached microalgae. The adhesion of the microalgae was observed using an inverted fluorescence microscope. The fluorescence image is shown in Figure S2.

Five fluorescence images were taken for each sample to determine the diatom anti-adhesion rate, which was then calculated using Equation (5) [29]. The anti-adhesion rate (R) was derived from Equation (5).

(5) $$R=\left(1-\frac{N_{4}}{N_{3}}\right)\times 100\%$$

The *P. tricornutum* count after water treatment of the PDMS coating was recorded as N_3_, while the *P. tricornutum* count after water treatment of the P-AM-x coating was documented as N_4_.

### 2.9. Marine Field Test

The field was conducted from 16 May 2023 to 12 September 2023 at the Xiamen Jimei University Aquatic Training Centre, China (24°56′ N, 118°10′ E). The samples were applied to epoxy resin plates (100 × 80 × 3 mm^3^) and immersed in seawater at a depth of 0.2 to 2.0 m. After a specified time, the coating was removed from the seawater, carefully cleaned with seawater and photographed for documentation purposes. Resistance was assessed according to the American Society for Testing and Materials (ASTM)D6990-05 (2011) [30]. The samples were then returned to seawater for further testing.

## 3. Results and Discussion

### 3.1. Chemical Characterization of P-AM Coatings

The preparation process of a silicone-based amphiphilic gel coating is shown in the Scheme 1c. PEG-MA was used as the hydrophilic monomer, while TPMA served as the crosslinking agent to generate a non-crosslinked polymer chain by radical polymerisation [24]. To mitigate excessive expansion of the hydrophilic polymers in aqueous environments, high-molecular-weight HO-PDMS-OH was incorporated. The PDMS, METES and non-crosslinked polymer chains were mixed together, followed by the hydrolysis of the silane to produce silanols. These silanols were then subjected to dehydration condensation to form the silicon-based amphiphilic gel coating. The FT-IR spectrum of AM is shown in Figure 1a and shows the presence of absorption peaks corresponding to C=O (1725 cm^−1^), -CH_3_ (1448 cm^−1^) and C-O-C (1145 cm^−1^). Furthermore, the absorption peaks at 1078 cm^−1^ and 816 cm^−1^ could be attributed to the Si-O-C and Si-C groups, respectively. Notably, no absorption peak associated with the C=C group (1637 cm^−1^) was observed in the spectra, providing evidence for the successful synthesis of AM polymers by free radical mechanisms. The chemical structure of AM was characterised by ^1^H NMR spectroscopy (Figure S1). δ 0.77–0.96 (Si-CH_2_) were the methylene proton absorption peaks of Si-CH_2_-CH_2_ in TPMA. δ 3.50–3.60 (Si-O-(CH_3_)_3_) was attributed to the proton peaks of the three methyl groups of the TPMA end group. δ 3.60–3.65 (O-CH_2_-CH_2_-O) were the methylene proton peaks on the PEG-MA segment [31]. The signals at δ 4.10–4.20 and δ 4.30–4.35 were assigned to the methylene protons (C-O-CH_2_) originating from TPMA and PEG-MA, respectively. Combining δ 1.88–1.97 (Si-CH_2_-CH_2_), δ 3.30–3.48 (O-CH_3_) and δ3.65–3.80 (O-CH_3_) [32,33], it is evident that the non-crosslinked polymer chain segment polymers were successfully prepared by radical polymerisation reactions with the three monomers. Subsequently, by introducing a minute amount of catalyst, the siloxane on the AM branched chain undergoes dehydration condensation with PDMS and METES, forming an amphiphilic crosslinked network by curing. Four different AM/PDMS ratios were prepared and characterised by ATR-IR (Figure 1b). The characteristic absorption peaks at 861 cm^−1^ and 1260 cm^−1^ indicate the Si-C stretching vibration, while the characteristic peak of -CH_3_ was shown at 2960 cm^−1^. Furthermore, the characteristic peak of the Si-O-Si group at 1003 cm^−1^ confirmed the occurrence of a condensation reaction during the curing process of the coating. Interestingly, with increasing AM addition, the characteristic absorption peak of ν_C=O_ can be observed at 1730 cm^−1^ on P-AM-15, indicating the successful formation of a co-network between AM and PDMS by condensation, accompanied by migration to the surface of the coating.

Amphiphilic polymers play a key role in the design of antifouling/foul release coatings. By strategically combining different monomers, one can achieve microphase separation and localised swelling within the coating, thereby creating a heterogeneous surface with a precise chemical composition at the micro–nano scale [34,35]. The surface morphology of the silicone-based amphiphilic gel coating was examined by SEM to determine its microscopic features (Figure 1c). Convex vesicles with a diameter of 1–4 microns were observed on the P-AM-5, P-AM-10 and P-AM-15 coatings compared to the PDMS coating. During the crosslinking reaction, phase separation occurred due to differences in hydrophilicity and hydrophobicity between the monomers, resulting in a thermodynamically unstable system where components with different properties tended to separate. However, macroscopic phase transitions were prevented by covalent bond limitations between different monomers occurring at the micro–nano scale, leading to the formation of convex vesicles on the surface. The silicon-based amphiphilic gel coating was immersed in ASW for 3 days and then observed by laser confocal microscopy to study its surface structure and stability. A comparison of the samples before and after immersion (Figure 1d) shows no significant changes in PDMS, P-AM-5 and P-AM-10. However, it is noteworthy that a large number of vesicle-like structures were observed on the surface of P-AM-15 before immersion, but their number decreased and changed to a densely packed mountain-like structure after immersion. As a result, a microdomain structure with more uniformly distributed hydrophilic and hydrophobic properties was formed. The research results suggest that this phenomenon can be attributed to surface reconstruction resulting from different degrees of chain segment movement exhibited by the hydrophilic and hydrophobic components within the amphiphilic coating under different environmental conditions. Furthermore, when comparing the Ra surface roughness of the samples before and after immersion (Table S1), it was observed that all the samples exhibited a change in roughness within 1 μm. The hydrophobic nature of PDMS prevents the coating from swelling due to excessive water absorption. In addition, TPMA acts as a strong crosslinking agent with other silanols, thereby limiting the expansion of the hydrophilic polymers on the surface of the coating. The demonstrated behaviour indicates that the coating exhibits exceptional resistance to swelling and deformation during the formation of a hydrated layer, ensuring its stability for use in marine environments.

**Figure 2:**
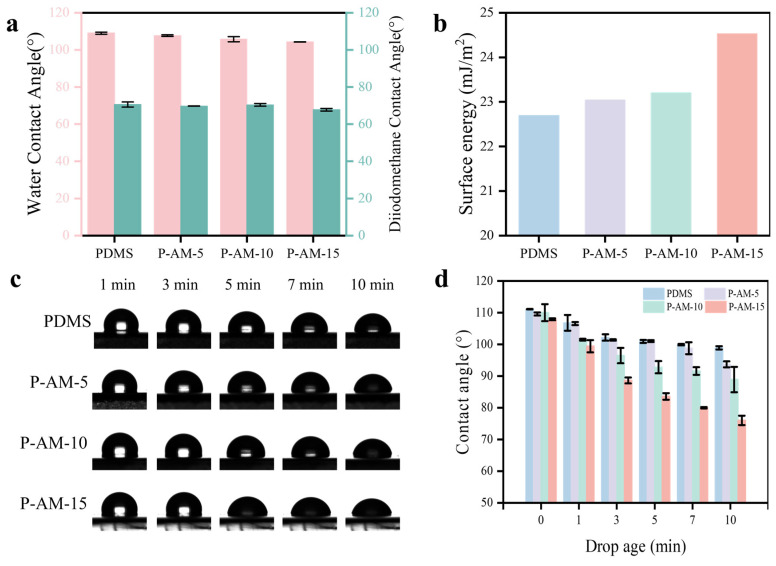
(**a**) Water and diiodomethane (CH_2_I_2_) contact angle tests of PDMS and P-AM-x coatings; the error bar represents the standard error derived from five independent replicates. (**b**) Surface free energy of PDMS and P-AM-x coatings. (**c**) The dynamic change process of water droplets and (**d**) the water dynamic contact angle of the coatings.

In this study, the wettability and surface energy of these coatings are investigated in Figure 2a,b. Figure 2a shows that the PDMS coating has a water contact angle of 108.99°. Its highly hydrophobic nature is attributed to the rapid orientation of methyl groups on the surface, resulting in a low surface tension and achieving a hydrophobic effect. The contact angle of the coating decreases slightly as the AM content increases. This phenomenon can be attributed to the preferential migration of high-molecular-weight PDMS towards the air–polymer interface during film formation, thereby maintaining the hydrophobicity of the coating. The surface energy of each sample was determined using the Owens–Winter method (Figure 2b). The γ^D^ and γ^P^ are summarised in Table S2. The surface energy of the coating is directly proportional to the content of polymer chain segments. It is known that γ^P^ is determined by the intermolecular forces between polar molecules in the material [27]. Increasing the content of the AM will result in a corresponding increase in γ^P^, thereby inducing an increase in surface energy. To further investigate the surface properties of the coating, the water dynamic contact angle was tested and shown in Figure 2c,d. Neither PDMS nor P-AM-5 show significant changes in the dynamic contact angle, with a value still above 90° after 10 min. Both P-AM-10 and P-AM-15 show a rapid decrease over time, particularly P-AM-15, which decreases from 89° to 74° in 3–10 min. The hydrophilic chain of P-AM-15 gradually migrates from the interior to the surface of the coatings after 3 min. This phenomenon means that the coating accumulates hydrophilic groups on its surface after immersion in seawater, forming a hydrated layer [28]. At the same time, PEG-MA exhibits remarkable anti-protein properties, reducing non-specific protein binding to the hydrophobic surface and giving the coating antifouling properties.

### 3.2. Antifouling Properties of the P-AM Coatings

#### 3.2.1. FITC-BSA Adsorption Tests

**Figure 3:**
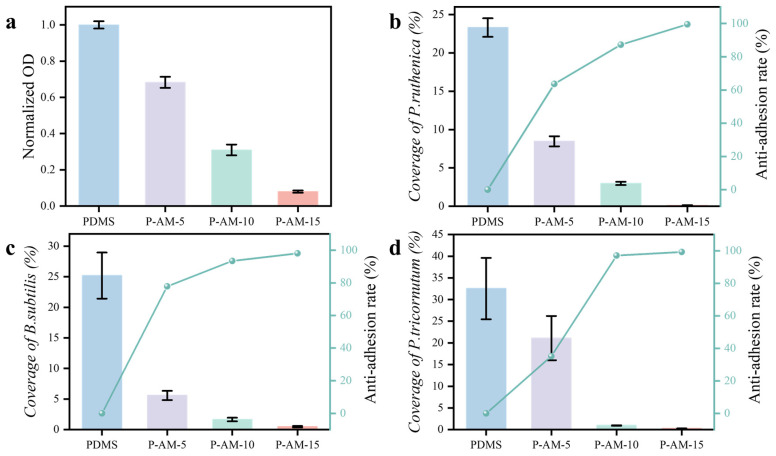
(**a**) Adhesion rate of FITC-BSA. Coverage and anti-adhesion rate of *P. ruthenica* (**b**), *B. subtilis* (**c**) and *P.tricornutum* (**d**).

Marine fouling is a continuous and stochastic process, typically initiated by the rapid accumulation of dissolved organic molecules in seawater, such as polysaccharides, proteins and lipids [27,36]. The hull surface generates abundant carbon sources that are used for reproduction by subsequent fouling organisms. Therefore, the representative FITC-BSA was chosen to assess the protein resistance of the coating. As shown in Figure 3a, the BSA adhesion rate on the PDMS surface is 1, and the protein adhesion rate on the coating surface is normalised, and the corresponding fluorescence image is shown in Figure S2. The PDMS coating surface showed a substantial amount of adhesion by FITC-BSA, whereas the adhesion on the P-AM-x surface was significantly reduced. Among the P-AM-x coatings, P-AM-15 in particular showed the lowest rate of surface protein adhesion. These results demonstrate that hydrophilic modification confers increased resistance to protein adhesion to the coating as a whole. As a typical hydrophobic material, PDMS exhibits non-specific protein binding [37], which can induce conformational changes and denaturation of proteins, facilitating their adhesion to surfaces. With the incorporation of hydrophilic polymers, PEG will form a compact hydration layer on the coating surface, effectively hindering physical protein adsorption and creating an energy barrier. The rapid migration of the hydrophilic component in P-AM-x under seawater immersion provides exceptional resistance to protein adhesion.

#### 3.2.2. Bacterial Adhesion Test

Biofilms of bacteria and organic matter form on the surface of materials exposed to the marine environment and are an essential factor in the adhesion of macrofouling organisms such as barnacles and mussels [38,39]. Therefore, improving the antibacterial adhesion of the coating can also improve the antifouling efficiency of the coating. In this paper, two representative marine bacteria, a Gram-positive bacterium (*B. subtilis*) and a Gram-negative bacterium (*P. ruthenica*), were selected to test the performance of the coating. By testing the anti-adhesion performance of four coating materials, we obtained data on the coverage and anti-adhesion rate of two bacteria on different coating surfaces (Figure 3b,c). The anti-adhesion rate was determined from the amount of adhesion observed on PDMS-coated surfaces. Specifically, *B. subtilis* was reduced by approximately 98.09%, while *P. ruthenica* was reduced by approximately 99.48% (Table S3). These results indicate that P-AM-x coatings have some antibacterial adhesion ability (the fluorescence image depicting bacterial adhesion is shown in Figure S2).

#### 3.2.3. Alga Resistance Tests

Microalgae are an integral part of the marine biological membrane. The biofilm is the early stage of the marine fouling process, followed by the topography of large organisms on the biofilm. Therefore, the representative *Phaeodactylum tricornutum* (*P. tricornutum*) was chosen as a focus of this research to study the adhesion of algae to the coating surface. Figure 3d shows the coverage and anti-adhesion rate of *P. tricornutum* cultivated on the coating surface for seven days after the washing treatment. The algae attachment area decreased for PDMS, P-AM-5, P-AM-10 and P-AM-15, with values of 32.53%, 21.11%, 0.95% and 0.25%, respectively, indicating excellent algae resistance when the amphiphilic P-AM content exceeds 10 wt% (the fluorescence image of surface adhesion is shown in Figure S2). In the initial phase of attachment, diatoms secrete extracellular polymers (EPS), composed of complex proteins and glycans, to adhere to the substrate surface. The EPS induces non-specific binding to PDMS, thereby increasing the adhesion strength of the diatoms. As an excellent anti-protein monomer, PEG-MA can effectively reduce protein adsorption and decrease diatom adhesion on P-AM surfaces. In addition, silicone-based amphiphilic gel coating improves the fouling release performance of ambiguous surfaces exposed to water [40,41,42].

#### 3.2.4. Marine Field Test

**Figure 4:**
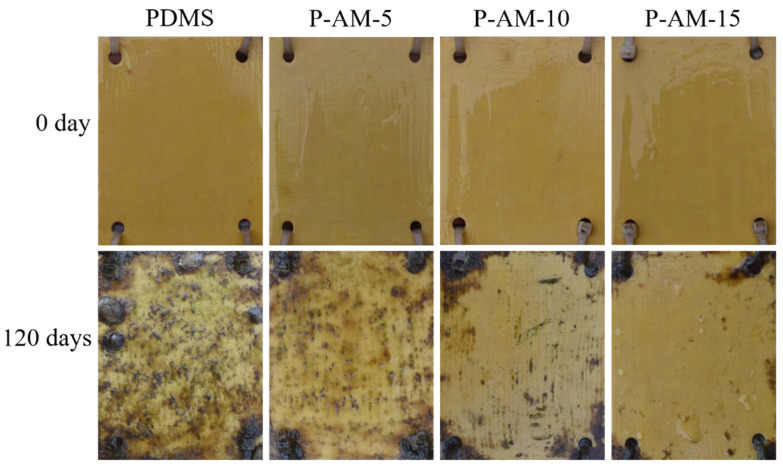
Figures of panels coated with the PDMS coating and the P-AM-X coating immersed in Xiamen (24°56′ N, 118°10′ E) for 0 days and 4 months.

The antifouling performance of the P-AM coatings was further evaluated in a marine environment (Xiamen, China, 24°56′ N, 118°10′ E). Figure 4 shows the photographic images of the PDMS and P-AM-X coatings before and after immersion in seawater for 4 months. The coatings showed excellent durability and adhesion throughout the test period, as evidenced by their intact surfaces, free of any damage or peeling. The PDMS coating was found to be heavily covered with sea silt, accompanied by the presence of fresh algae, barnacles and their larvae. The surface of P-AM-5 showed a significant reduction in marine silt adhesion and a reduction in barnacle and algae adhesion. The surface of P-AM-10 showed no apparent fouling organisms, although there were minor accumulations of algae and marine sediment. The surface of the P-AM-15 coating showed minimal fouling organisms and only a negligible amount of marine silt deposition, indicating its exceptional antifouling biological properties. These results further demonstrate that the amphiphilic modification of PDMS can effectively enhance the antifouling performance of silicone coatings, giving them long-term potential for marine antifouling.

**Figure 5:**
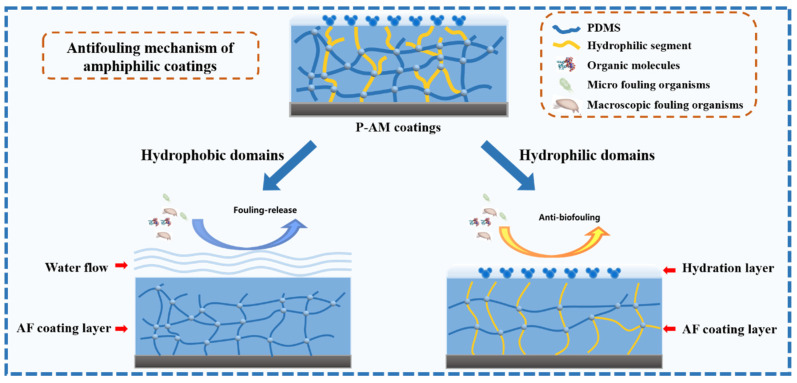
Antifouling mechanisms of P-AM coatings against fouled organisms.

Based on the above experimental results, a potential mechanism can be proposed to explain the antifouling performance of the coating (Figure 5):

The surface of amphiphilic coatings consists of both hydrophobic and hydrophilic components that undergo surface reconstruction in the marine environment. Combined with their inherent hydrophilic and hydrophobic properties, they form a unique “mosaic” blurred surface structure [20,28]. This distinctive surface architecture enables the silicone-based amphiphilic gel coating to exhibit an excellent antifouling performance. At the same time, the coating retains the foul release (FR) properties of PDMS, while also possessing the foul resistance properties of the hydrated hydrophilic layer. In marine environments, the rapid formation of a water layer at the seawater interface by the hydrophilic structural domains on the surface prevents adhesion proteins or extracellular polymers produced by fouling organisms from adhering to the surface of the coating. During ship operation, shear erosion caused by seawater helps to effectively remove fouling organisms adhering to the surface, thereby achieving efficient fouling release.

## 4. Conclusions

We present a silicone-based amphiphilic co-network coating with excellent antifouling and underwater surface restoration properties. TPMA was used as the crosslinking agent to form a covalently bonded network of hydrophilic and hydrophobic regions, effectively preventing macroscopic phase separation between the two incompatible components. In the amphiphilic coating, hydrophilic polymers undergo rapid migration to the surface in an underwater environment, resulting in structural changes to the surface. A dense hydration layer is formed with water molecules through hydrogen bonding, which further enhances the resistance to protein, bacterial and algal adhesion. Our experimental results show the excellent antifouling properties of this coating. Due to its exceptional properties and unique surface characteristics, our proposed amphiphilic co-network coating has great potential for marine antifouling applications and contributes to the advancement of amphiphilic polymer materials.

## Data Availability

The original contributions presented in this study are included in the article/supplementary material; further inquiries can be directed to the corresponding authors.

## Supplementary Materials

The following supporting information can be downloaded at: https://www.mdpi.com/article/10.3390/polym16111570/s1, Figure S1: ^1^H NMR of AM; Figure S2: The adhesion fluorescence images of FITC-BSA, *B. subtilis*, *P. ruthenica*, and *P. tricornutum*; Table S1: The roughness (Ra and Rq) of the coating before and after immersion in seawater for 3 days; Table S2: Contact angles (θ) and Suface Energy on the coating surfaces; Table S3: The proportion of coating content, the coverage of typical fouling substances (protein, bacteria, algae) on the coating surface, and the anti-adhesion rate.
