# Supplementary material for: Surface Reconstruction of Silicone-Based Amphiphilic Polymers for Mitigating Marine Biofouling
Source: Polymers (Basel). 2024 Jun 1;16(11):1570. doi: 10.3390/polym16111570 (PMC11174759; doi:10.3390/polym16111570)
Supplement: Supplementary file 1 [file polymers-16-01570-s001.zip › polymers-2997820-supplementary.pdf]

Supporting Information

Surface reconstruction of silicone-based amphiphilic polymers for mitigating marine biofouling

Chuanying Wei[1], Yan Zhang[1], Zhen Tang[1], Changan Zhang[1], Jianhua Wu[1*], Bo Wu[1*]

[1] Xiamen Key Laboratory of Marine Corrosion and Intelligent Protection Materials, JiMei University, Xiamen, Fujian 361021, China

* Corresponding author: Bo Wu: wubo@jmu.edu.cn; Jianhua Wu: wujh@jmu.edu.cn.

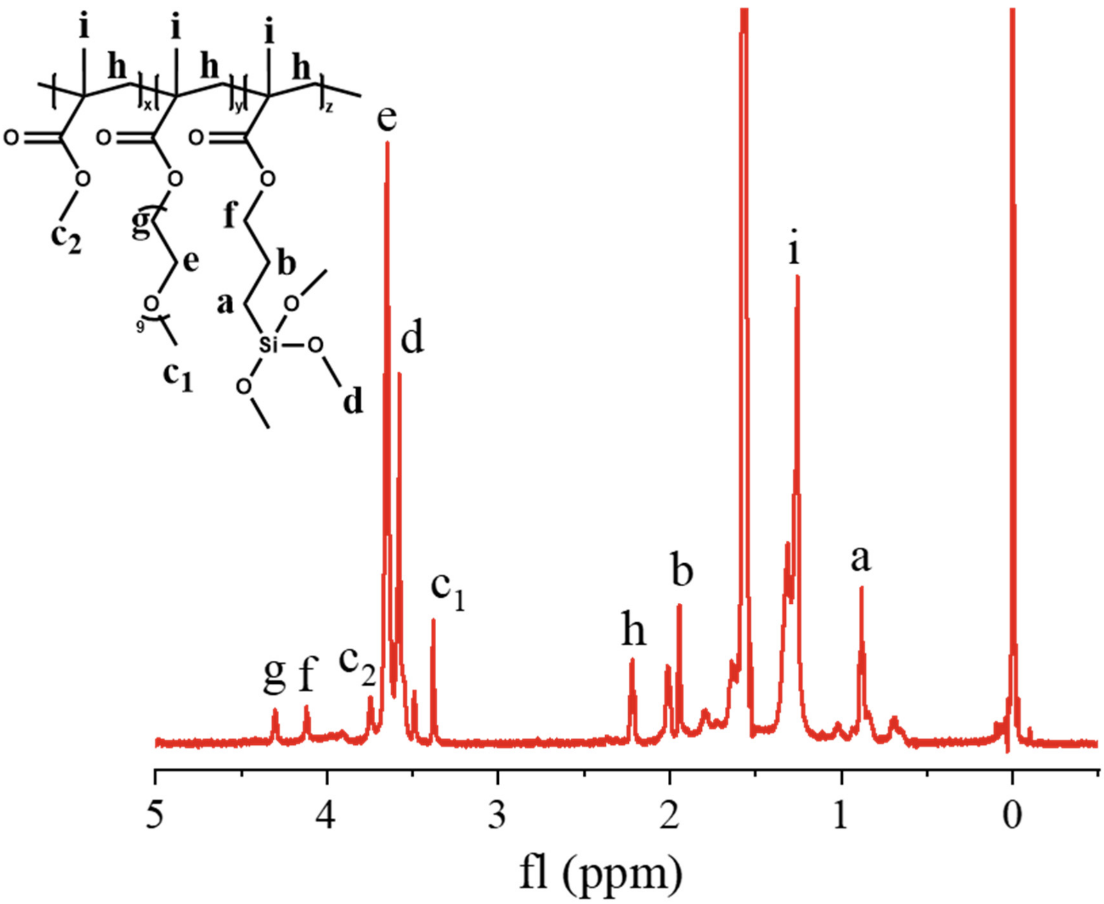

Figure S1. ^{1}H NMR of AM. ^{1}H NMR ($CDCl_3$, δ ppm): 0.77-0.96 (Si-<u>CH2</u>); 1.15-1.46 (<u>CH3</u>-C); 1.88-1.97 (Si-CH_2-<u>CH2</u>); 2.17-2.28 (C-<u>CH2</u>); 3.30-3.48, 3.65-3.80 (O-<u>CH3</u>); 3.50-3.60 (Si-O-(<u>CH3</u>)$_3$); 3.60-3.65 (O-<u>CH2</u>-<u>CH2</u>-O); 4.10-4.20 and 4.30-4.35 (C-O-<u>CH2</u>) [1,2].

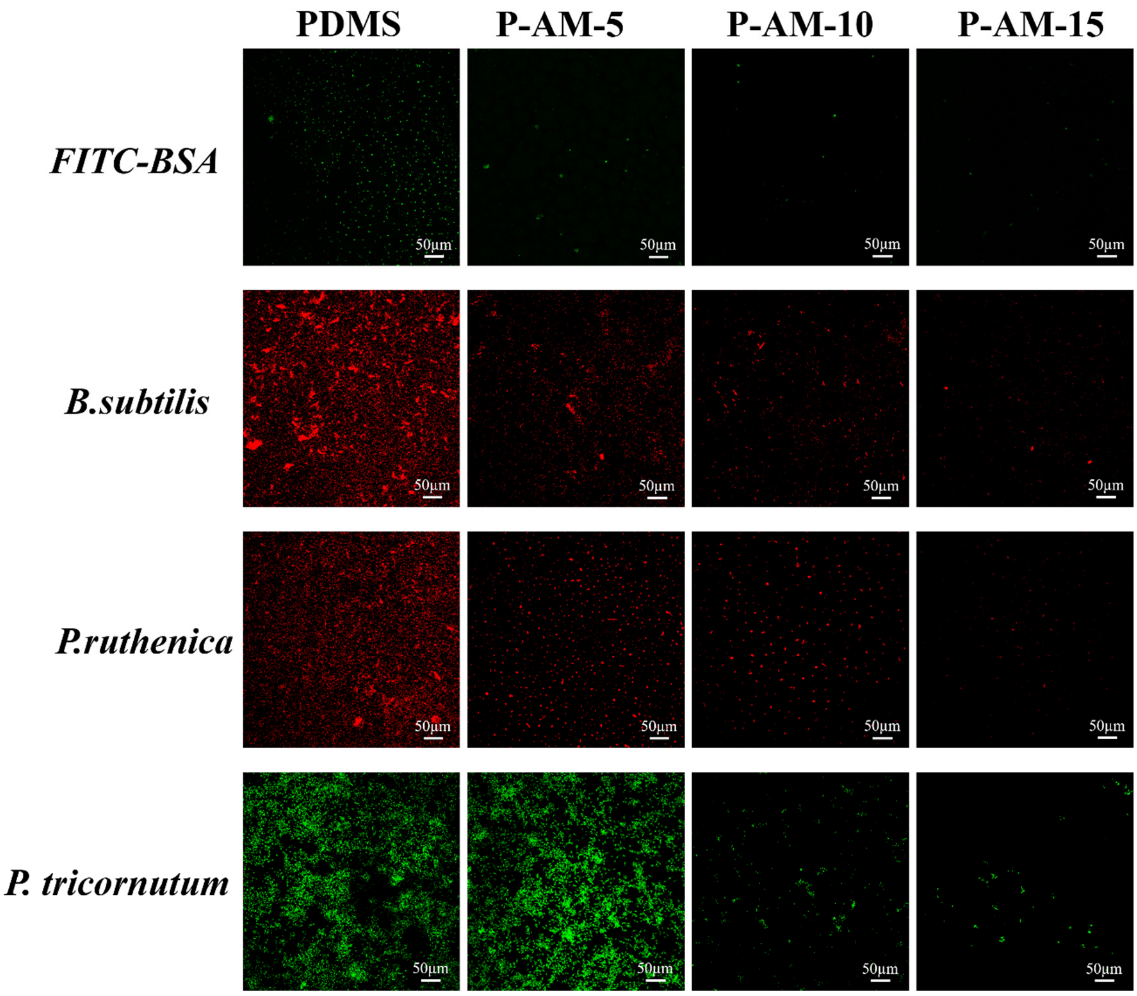

Figure S2. The adhesion fluorescence images of *FITC-BSA*, *B.subtilis*, *P.ruthenica*, and *P.tricornutum*.

Table S1. The roughness (Ra and Rq) of the coating before and after immersion in seawater for 3 days.

Sampels	ASW immersion	Ra(μm)	Rq(μm)
PDMS	Before	1	2
	After-3 days	2	3
P-AM-5	Before	2	2
	After-3 days	2	3
P-AM-10	Before	2	3
	After-3 days	3	4
P-AM-15	Before	3	4
	After-3 days	4	5

Table S2. Contact angles (θ) and Suface Energy on the coating surfaces.

Sampels	θ (◦)		Suface Energy（mJ/m²）		
	Water	Diiodomethane	γ^D	γ^P	γ^S
PDMS	108.99±0.61	70.53±1.36	22.58	0.11	22.69
P-AM-5	105.73±0.46	70.35±0.05	22.68	0.36	23.04
P-AM-10	107.61±1.39	69.73±0.75	23.03	0.17	23.20
P-AM-15	104.27±0.05	67.76±0.70	24.13	0.39	24.53

Data are referred from ISO 19403-2:2017《PAINTS AND VARNISHES - WETTABILITY - PART 2: DETERMINATION OF THE SURFACE FREE ENERGY OF SOLID SURFACES BY MEASURING THE CONTACT ANGLE》

Table S3. The proportion of coating content, the coverage of typical fouling substances (protein, bacteria, algae) on the coating surface, and the anti-adhesion rate.

Sample	Content of PDMS （g）	Content of AM（g）	Coverage（%）			Anti-adhesion rate (%)		
			B.subtilis	*P.ruthenica*	*P. tricornutum*	*B.subtilis*	*P.ruthenica*	*P. tricornutum*
PDMS	5	0	25.19	23.30	32.53	—	—	—
P-AM-5	5	0.275	5.59	8.47	21.11	77.81	63.65	35.11
P-AM-10	5	0.55	1.65	2.97	0.95	93.45	87.25	97.08
P-AM-15	5	0.825	0.48	0.12	0.25	98.09	99.48	99.23

References

1. Q, Zhang.; J, Hong.; R, Hoogenboom. A triple thermoresponsive schizophrenic diblock copolymer. *Polym. Chem* **2013**, *4*, 4322.
2. M, Matsumoto.; M, Takenaka.; M, Sawamoto.; T, Terashima. Self-assembly of amphiphilic block pendant polymers as microphase separation materials and folded flower micelles. *Polym. Chem* **2019**, *10*, 4954.
